# CatDive: A simple yet effective method for maximizing category diversity in sequential recommendation

**DOI:** 10.1371/journal.pone.0341419

**Published:** 2026-01-30

**Authors:** Jaeri Lee, Jongjin Kim, U. Kang

**Affiliations:** Data Mining Lab, Seoul National University, South Korea; University of Cape Town, SOUTH AFRICA

## Abstract

How can we offer users items of diverse categories while considering their interests in sequential recommendation? Traditional sequential recommendation methods often prioritize accuracy over diversity, recommending only items similar to those with which users have previously interacted. Recommendation biased toward only the favorite category of each user fails to achieve novelty and serendipity, demoting users’ satisfaction. Thus, it is important to maximize category diversity in sequential recommendation. However, the state-of-the-art work on category diversified sequential recommendation tackles it only with a post-processing method which sacrifices accuracy to improve category diversity. In this paper, we propose CatDive, a simple yet effective method for maximizing category diversity without sacrificing accuracy in sequential recommendation. In the training phase, CatDive incorporates category information into the recommendation model by using multi-embedding, composed of item and category embeddings. Also, CatDive boosts category diversity by placing greater emphasis on users’ preferred categories when selecting negative samples, while still prioritizing high-confidence negatives based on item popularity to maintain accuracy. Finally, we enhance and control category diversity by prioritizing underrepresented categories in the reranking phase, using a tunable hyper-parameter to balance adjustments. CatDive achieves the state-of-the-art performance on real-world datasets, achieving up to 152.49% higher category diversity in the similar level of accuracy and up to 39.73% higher accuracy in the similar level of category diversity compared to the best competitor.

## Introduction

*How can we offer users items of diverse categories while considering their interests in sequential recommendation?* In an era where data are abundant and user choices are vast, sequential recommender systems have emerged as a critical tool in various domains, from e-commerce to streaming services, aiding users in their decision-making processes [1]. Unfortunately, traditional sequential recommendation methods often prioritize accuracy over diversity, tending towards recommending only items similar to those with which users have previously interacted. This practice leads to a phenomenon known as the “filter bubble [2],” wherein users are continuously exposed to a limited range of items or categories. It restricts novelty and serendipity, two elements that significantly contribute to enhancing users’ experience and satisfaction. One of the solutions to this unwanted phenomenon is category diversified sequential recommendation. By introducing interesting items in new categories to users, it aims to enhance discovery, increase the long-term engagement of users, and break free from the constraints of the filter bubble.

**Fig 1:**
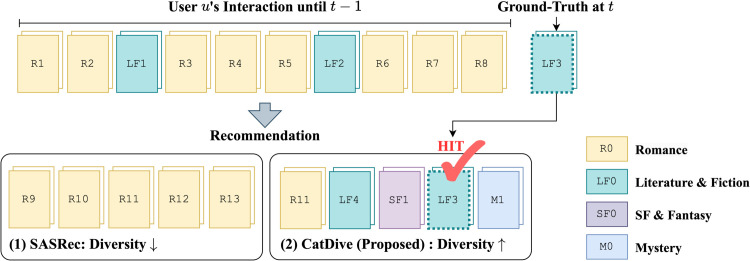
A real-world example of category-diversified and accurate recommendation of CatDive. Each rectangle represents a book; its color indicates the category, and the number inside denotes the book index. CatDive recommends items of diverse categories and also predicts the ground-truth item, while SASRec fails, recommending items only in the user’s favorite category.

However, limited studies on category diversified sequential recommendation have been done, and traditional sequential recommendation models [3–5] focus only on the accuracy of the recommendation. Recommendation (1) in the dotted box of Fig 1 depicts a real-world example of recommendation in Amazon Books created by a sequential recommendation model SASRec [6] given a user’s history sequence. Books with borders of the same color are in the same category. For instance, books with yellow borders are all romance books. SASRec focuses heavily on the romance category which appeared in the user’s history sequence the most frequently. As a result, it recommends all items from the romance category only, failing to predict the ground-truth item which is in the literature & fiction category. Moreover, the state-of-the-art work [7] on category diversified sequential recommendation leverages only a post-processing method, failing to achieve high category diversity while maintaining accuracy.

In this work, we propose CatDive (Category Diversified Sequential Recommendation), a simple yet effective recommendation method for maximizing category diversity without sacrificing accuracy in sequential recommendation. During training, CatDive integrates category information into the recommendation model through a multi-embedding structure, combining both item and category embeddings. Furthermore, CatDive enhances category diversity by assigning greater importance to users’ preferred categories when selecting negative samples, while still giving priority to high-confidence negatives based on item popularity to preserve accuracy. Finally, we improve and regulate category diversity in the reranking phase by prioritizing underrepresented categories, using a tunable hyper-parameter to balance diversity and accuracy. Recommendation (2) in the dotted box of Fig 1 depicts the recommendation of CatDive given the same user’s history. CatDive recommends items of diverse categories, including the ground-truth item in a less preferred category.

Our contributions are summarized as follows:

- **Method.** We propose CatDive, a simple but effective approach for category diversified sequential recommendation. CatDive exploits multi-embedding, preference-confidence based negative sampling, and coverage-prioritized reranking to maximize category diversity while maintaining high accuracy.
- **Generality.** We show that CatDive is generally applicable for existing sequential recommendation models. CatDive improves both category diversity and accuracy when applied to different models.
- **Experiments.** Extensive experiments show that CatDive achieves up to 152.49% higher category diversity in the similar level of accuracy and up to 39.73% higher accuracy in the similar level of category diversity in top-*k* sequential recommendation compared to the best competitor, resulting in the state-of-the-art performance.

In the rest of our paper, we provide the related works, introduce our proposed method, evaluate the proposed method and competitors on real-world datasets, and conclude this work. The code and datasets are available at https://github.com/snudatalab/CatDive. Table 1 presents notations frequently used in this paper.

**Table 1:** Table of symbols.

Symbol	Terminology	Description
ℐ	item set	the set of items in a dataset
${ℐ}_{u}$	user item set	the set of items interacted by user *u*
𝒰	user set	the set of users in a dataset
𝒞	category set	the set of categories in a dataset
*c*(*i*)	category	category of item *i*
${𝐡}^{u}$	interaction history	list of items that user *u* has interacted with
*k*	recommendation size	number of items recommended to each user
${ℛ}_{u}^{k}$	recommendation	top-*k* recommendation given to user *u*
${𝐞}_{i}^{it}$	item embeddings	embeddings for item *i*
${𝐞}_{c}^{cat}(i)$	category embeddings	embeddings for category *c*(*i*)
${𝐞}_{i}^{m}$	multi-embeddings	concatenation of item embeddings and category embedding for item *i*
*int*(*u*,*i*)	user-item interactions	the number of interactions between user *u* and item *i*
*int*(*u*,*c*(*i*))	user-category interactions	the number of interactions between user *u* and item *i*’s category *c*(*i*)
$s_{u,i}^{\text{pref}}$	category-preference score	the score of item *i* being selected as a negative sample for user *u* considering the user’s category preference
$s_{i}^{\text{conf}}$	confidence score	the score of item *i* being selected as a negative sample considering the item’s confidence
*s*_*u*,*i*_	negative sampling score	the score of item *i* being selected as a negative sample for user *u*
*α*	category weight	a weight to control the impact of the category embedding
*β*	confidence weight	a weight to control the effect of confidence score
$\lambda_{1}$	coverage weight	a weight to control the degree of coverage ensurance in coverage-prioritized reranking
$\lambda_{2}$	penalty weight	a weight to control the degree of penalty in coverage- prioritized reranking

## Related work

### Sequential recommendation

Sequential recommendation is a subfield of recommender systems that focuses on predicting what item a user will interact with next, given their past sequence of interactions. This type of system is particularly useful in domains where the order and timing of events matter, such as music streaming, online shopping, or OTT (Over The Top) services. One common approach for building sequential recommendation systems is using Recurrent Neural Networks (RNNs), which are particularly well-suited for handling sequential data. GRU4Rec [6] is a well-known model based on RNN, which modifies classic RNNs to make them more suitable for the sequential recommendation problem. Other methods like Markov Chains [8,9] or more recent Transformer-based models [10,11] are also used depending on specific use cases and requirements. One of the most recent and state-of-the-art models, SASRec [12], is based on a self-attention mechanism that allows it to capture both short and long-term semantics of user interaction history. Sequential recommendation differs from the problem of category diversified sequential recommendation since the former ignores the diversity of categories in recommendation, which is required for improving users’ experience. In this work, we propose a category diversified sequential recommendation method outperforming previous works.

### Diversified recommendation

Diversified recommendation focuses on giving a variety of items in recommendation to produce diverse results while sacrificing the minimum accuracy. Recently, diversified recommendation has been studied to enhance user experience and maximize platform revenue [13]. There are two kinds of diversified recommendations: aggregate-level and individual-level.

#### Aggregate-level diversified recommendation.

Aggregate-level diversified recommendation aims to increase diversity across the entire set of recommendations for all users [14–16]. Achieving high overall diversity is crucial as it helps mitigate issues associated with the long tail effect and enhances the profitability of a sales platform. Many current techniques for generating recommendations with high overall diversity involve adjusting the output of a primary model, since optimizing the model for both accuracy and diversity is a difficult and challenging task.

#### Individual-level diversified recommendation.

Individual-level diversified recommendations aim to enhance the user experience by presenting a varied set of items to each user, thus catering to a wider range of their interests and preferences [13,17]. By targeting diversity at the individual level, recommendation systems can provide more engaging and satisfying content, potentially increasing user satisfaction and platform engagement.

Category diversified sequential recommendation belongs to individual-level diversified recommendation since it aims to diversify the category distribution within a single user’s recommendation list. However, in previous studies on individual-level diversified recommendation, there has been a limited attempt to utilize the sequence information of user interactions [18]. Category diversified sequential recommendation is different from item-level diversified recommendation since the former leverages users’ sequence information and focuses on the diversification of categories rather than that of items. CatDive leverages a sequential recommendation model to consider the sequence information in category diversified recommendation.

## Proposed method

We formally define the problem of category diversified sequential recommendation and propose CatDive, a simple yet effective method for the problem.

### Category diversified sequential recommendation

Category diversified sequential recommendation ensures that the items suggested to a user span a variety of categories or genres in sequential recommendation. The key challenge with category diversified sequential recommendation lies in finding an optimal balance between accuracy and category diversity. Accuracy refers to how closely the recommended items align with a user’s known preferences. Category diversity, on the other hand, involves introducing users to new categories or genres that they might find interesting based on their past behavior and similar users’ behavior. Too much emphasis on accuracy could lead to overspecialization where users are recommended items from only one category or genre, limiting their exposure to potentially interesting content from other categories. On the flip side, too much emphasis on category diversity could result in recommending items that are too far from the user’s preferences, leading to lower satisfaction.

The state-of-the-art work on category diversified sequential recommendation, ComiRec [7], controls the category diversity using a post-processing method that reranks candidate items. Unfortunately, post-processing methods are limited to selecting items from an already chosen item pool, eventually sacrificing accuracy when trying to increase category diversity.

The problem of category diversified sequential recommendation is defined formally as follows:

**Definition 1.** *Given a user u, an item set* $ℐ=\{i_{1},i_{2},\ldots ,i_{\left|I\right|}\}$*, a category set* $𝒞=\{c(i_{1}),c(i_{2}),\ldots ,c(i_{\left|ℐ\right|})\}$ *of items, and the user’s item interaction history* ${𝐡}^{u}=\{i_{1}^{u},i_{2}^{u},\ldots ,i_{t-1}^{u}\}$ *until timestamp t–1, the goal of category diversified sequential recommendation is to recommend top-k items* ${ℛ}_{k}^{u}$ *that the user u will interact on timestamp t, such that the recommendation* ${ℛ}_{k}^{u}$ *is accurate and categories* $c(i\in {ℛ}_{k}^{u})$ *of items are diverse.*

The category diversity is evaluated by the following two metrics:

- **Intra-List Distance@*k* (ILD@*k*)**:
  $$\text{ILD}@k=\frac{\sum_{i,j\in {ℛ}_{k}^{u},i\neq j}\delta (c(i)\text{⧸}=c(j))}{k\times (k-1)},$$ (1)
  where *k* is the number of items recommended, *c*(*i*) is the category of item *i*, ${ℛ}_{k}^{u}$ is the set of top-*k* recommended items for user *u*, and *δ* is an indicator function. ILD@*k* represents how many pairs of items have different categories in the recommendation list of the user. It ranges from 0 to 1, and it is 1 if all categories of items in the recommendation list are distinct.
- **Coverage@*k* (cov@*k*)**:
  $$\text{cov}@k=\frac{\left|\{c(i)|i\in {ℛ}_{k}^{u}\}\right|}{\left|𝒞\right|},$$ (2)
  where |𝒞| is the number of existing categories. cov@*k* represent how many different categories appear in the recommendation list of the user. It ranges from 0 to 1, and 1 means that the recommendation covers all categories.

### Overview

We address the following challenges to achieve a high performance in category diversified sequential recommendation:

C1. **Reflecting Category Information in a Backbone Model.** To recommend items considering the category of items, a model needs to recognize the category information. How can we reflect category information in a backbone model?
C2. **Navigating the Trade-off Between Category Diversity and Accuracy during Model Training.** During the training process, a user embedding overfits to items within the user’s preferred categories, resulting in recommendations that lack diversity and focus too narrowly on those categories. Yet, to maintain accuracy, the embeddings must still closely align with the user’s true preferences, creating a trade-off between diversity and accuracy. How can we simultaneously increase both category diversity and accuracy during the training process of a backbone model?
C3. **Overcoming the Constraints of Diversity Control.** Training imposes inherent limitations that prevent us from freely achieving the desired level of category diversity. How can we attain a broader range of diversity with the least possible impact on accuracy after the training of a model?

The main ideas of CatDive are summarized as follows:

I1. **Multi-embedding.** We create category embeddings in addition to item embeddings, and jointly learn both embeddings to exploit category information in the backbone model.
I2. **Preference-Confidence Based Negative Sampling.** During training, we sample more negative items from the user’s preferred categories to prevent the user representation overfitting on those categories, while also prioritizing high-confidence negatives based on item popularity to maintain accuracy.
I3. **Coverage-Prioritized Reranking.** We enhance and control category diversity by prioritizing underrepresented categories in the reranking process, with minimal sacrifice to accuracy, using a tunable hyper-parameter.

**Fig 2:**
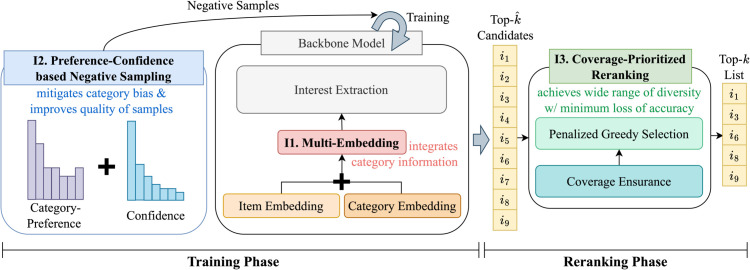
Overview of CatDive, which consists of training and reranking phases.

Fig 2 shows the overall process of CatDive. CatDive consists of two phases, model training phase and reranking phase. In the model training phase, CatDive first enhances the backbone model by replacing the traditional item embedding with a multi-embedding approach that leverages category information. Then, CatDive trains the model with negative samples selected according to the probability formed by preference-confidence based negative sampling, improving both category diversity and accuracy. In the reranking phase, CatDive improves and controls category diversity by prioritizing underrepresented categories with minimal impact on accuracy, using a tunable hyper-parameter to adjust the extent of diversity enhancement. These ideas of CatDive are applicable to various recommendation models as shown in experiments.

### Multi-embedding

How can we reflect the category information of items in the backbone model? In many previous models, item embeddings are randomly initialized for each item and then trained. However, this does not reflect the category information of items, and is not meaningful in obtaining category diversity.

Our idea is to create the embeddings to represent items using category information. That is, we additionally create category embeddings ${𝐞}^{\text{pref}}\in {\mathbb{R}}^{|𝒞|\times d}$ with the item embeddings ${𝐞}^{it}\in {\mathbb{R}}^{|ℐ|\times d}$ and add them to represent items as multi-embeddings, where |𝒞| is the number of categories in the category set 𝒞, |ℐ| is the number of items in the item set ℐ, and *d* is the embedding dimension. The category embeddings are randomly initialized for each category and then trained by the model. The multi-embedding ${𝐞}_{j}^{m}\in {\mathbb{R}}^{d}$ for item *j* is as follows:

$${𝐞}_{j}^{m}={𝐞}_{j}^{it}+\alpha \cdot {𝐞}_{c(j)}^{\text{pref}}$$ (3)

where ${𝐞}_{j}^{it}\in {\mathbb{R}}^{d}$ is the item embedding of item *j*, ${𝐞}_{c(j)}^{\text{pref}}\in {\mathbb{R}}^{d}$ is the category embedding of category *c*(*j*), and *α* is a hyper-parameter to control the impact of the category embedding.

### Preference-confidence based negative sampling

How can we simultaneously increase both category diversity and accuracy during the training process of the backbone model? We propose a comprehensive negative sampling strategy that integrates two key components, category weights and confidence of the samples, which mitigate category bias and improves accuracy, respectively.

In the training phase of a recommendation model, negative sampling strategy is widely used to learn from implicit feedback. Its goal is to provide negative instances that help the model distinguish between favored and unfavored items. To this end, unobserved user–item interactions are regarded as potential negatives, and a subset of them is sampled. This process is typically used in conjunction with loss functions such as Bayesian Personalized Ranking (BPR) and Sampled Softmax (SS) loss. The BPR and SS loss are defined as follows:

$${ℒ}^{\text{BPR}}=\sum_{(u,i^{+},i^{-})\in D}-\ln\sigma \left({\hat{y}}_{u,i^{+}}-{\hat{y}}_{u,i^{-}}\right),\;{ℒ}^{\text{SS}}=-\log\frac{\exp({\hat{y}}_{u,i^{+}})}{\exp({\hat{y}}_{u,i^{+}})+\sum_{i^{-}\in N_{u}}\exp({\hat{y}}_{u,i^{-}})},$$ (4)

where ${\hat{y}}_{u,i}$ represent the predicted scores of the model for user *u* on item *i*. The set *D* contains tuples $\left(u,i^{+},i^{-}\right)$ where *i*^+^ is a positive item that user *u* has interacted with and *i*^−^- is a negative item sampled from the unobserved data. *N*_*u*_ is the set of negative samples for user *u*. The primary objective of these losses is to assign higher scores to positive samples and lower scores to negative samples. Consequently, items selected as negative samples are less likely to be recommended by the model.

A naive approach to negative sampling involves randomly selecting items that the user has not interacted with. However, random sampling leads to selecting negative samples primarily from items in less preferred categories of each user, which results in the model recommending only items from the user’s preferred categories, thus reducing diversity. Additionally, when negative samples are drawn from lesser-known items that the user might not have encountered, it causes the model to incorrectly assume the user dislikes those items, thereby reducing the overall accuracy of the recommendations.

To address the challenge of random negative sampling, we propose an effective approach that evaluates the suitability of each item as a negative sample using a scoring function *s*_*u*,*i*_ and select negative samples according to the score. Thus, the probability P(i|u) of selecting an item $i\in {ℐ}_{u}^{-}$ as a negative sample for user *u* based on the score *s*_*u*,*i*_ as follows:

$$P(i|u)=\frac{s_{u,i}}{\sum_{i\in {ℐ}_{u}^{-}}s_{u,i}},$$ (5)

where ${ℐ}_{u}^{-}=ℐ\;⧵\;{ℐ}_{u}$ is the set of all potential negative samples for user *u*. This probability distribution ensures that items with higher scores are more likely to be selected as negative samples. In practice, when negative samples are pre-generated before training, the sampling process introduces no additional time overhead during the training phase.

We next define the scoring function *s*_*u*,*i*_ that quantifies the suitability of each item as a negative sample. The scoring function *s*_*u*,*i*_ integrates category-preference and confidence score into a unified scoring function. It is defined as:

$$s_{u,i}=s_{u,i}^{\text{pref}}+\beta \cdot s_{i}^{\text{conf}},$$ (6)

where $s_{u,i}^{\text{pref}}$ is the category-preference score of item *i* for user *u* and $s_{u,i}^{\text{conf}}$ is the confidence score of item *i*. *β* is a hyper-parameter to control the level of adjustment; a larger *β* gives more weight to $s_{i}^{\text{conf}}$. Although *β* is global in our current setup, the framework is extensible to per-user $\beta_{u}$, which adjusts the confidence according to user-specific preferences and features. Category-preference score $s_{u,i}^{\text{pref}}$ ensures the category-diversity of recommendation while confidence score $s_{u,i}^{\text{conf}}$ improves the accuracy of recommendation. The specific definitions of the category-preference score $s_{u,i}^{\text{pref}}$ and confidence score $s_{u,i}^{\text{conf}}$ are discussed in detail in the following subsections.

#### Category-preference score.

How can we prevent the model from recommending the items only in preferred categories? Our idea is to sample negative items proportionally to each user’s category preference. Through this, we avoid the overfitting of the user embedding to the preferred categories’ items. Randomly sampling from them results in more skewed recommendation towards preferred category, leading to low diversity. Instead, by selecting more items in preferred category as negative samples, the resulting recommendation becomes not skewed to the preferred category.

To achieve this, we design the category-preference score $s_{u,i}^{\text{pref}}$ and select negative samples that align with the user’s category preference. Based on this objective, we define the following desired property for score $s_{u,i}^{\text{pref}}$:

**Property 1.** $s_{u,i}^{\text{pref}}>s_{u,i^{\prime }}^{\text{pref}}$ *if* $int(u,c(i))>int(u,c(i^{\prime }))$*, where int(u,x) is the number of interaction between user u and x which is either an item or a category.*

Property 1 states that a preferred category should be given a higher priority for negative samples than a less preferred category. We define the score $s_{u,i}^{\text{pref}}$ as follows:

$$s_{u,i}^{\text{pref}}=\frac{int(u,c(i))}{\sum_{j\in {ℐ}_{u}}int(u,c(j))},$$ (7)

where ${ℐ}_{u}$ is the set of items that user *u* has interacted with. It is straightforward to show that Eq (7) satisfies the Property 1, which is summarized in Lemma 1.

**Lemma 1.** Eq (7) *satisfies Property 1.*

$s_{u,i}^{\text{pref}}$ is added in the final negative sampling score to select negative samples for user *u*, thus an item with a higher $s_{u,i}^{\text{pref}}$ is more likely to be selected as a negative sample (see Eq (6)).

#### Confidence score.

How can we maintain the accuracy of recommendation while improving the category diversity? Note that in the preferred categories of a user, there are possibly positive items as well as negative items. Thus, we have to carefully select negative items from them by finding high-confidence negative items that are likely to be negative items.

Our main idea to select high-confidence negative items is to consider the popularity of items. Recall that negative items should be selected among items that the user has not interacted with. Assume that a user *u* has not interacted with an item *i*. There are two possible reasons for this. First, *u* may not know the existence of *i*. Second, *u* does not like *i* even though *u* knows *i*. Then, it makes sense to select a negative sample that corresponds to the second case (*u* does not like *i* even though *u* knows *i*), since it is not clear whether *u* dislikes *i* in the first case.

Based on this idea, we design the score $s_{i}^{\text{conf}}$, which ensures that items with higher popularity are more likely to be selected as negative samples. The score $s_{i}^{\text{conf}}$ should satisfy the following property:

**Property 2.** $s_{i}^{\text{conf}}>s_{i^{\prime }}^{\text{conf}}$ *if* $\sum_{u\in 𝒰}int(u,i)>\sum_{u\in 𝒰}int(u,i^{\prime })$*, where* 𝒰 *is the set of users.*

Property 2 states that a popular item should be given a higher priority for negative samples. We show in the ablation study that giving such higher priority to a popular item is effective for finding high-confidence negative items and boosting accuracy. We define the scoring function $s_{i}^{\text{conf}}$ as follows:

$$s_{i}^{\text{conf}}=\frac{\sum_{u\in 𝒰}int(u,i)}{\sum_{u\in 𝒰,j\in ℐ}int(u,j)},$$ (8)

where ℐ is the set of all items. It is clear that Eq (8) satisfies Property 2, which is summarized in Lemma 2. We denote $\sum_{u\in 𝒰}int(u,i)$ and $\sum_{u\in 𝒰,j\in ℐ}int(u,j)$ as int(𝒰,i) and int(𝒰,ℐ) for brevity, respectively.

**Lemma 2.** Eq (8) *satisfies Property 2.*

### Coverage-prioritized reranking

How can we attain a broader range of diversity with the least possible impact on accuracy after the training of the model? Existing reranking algorithms [7,19] apply penalties to items from the same category and select items in a greedy manner. However, since similar items tend to have close ranks, items from the same category often have similar scores. As a result, such minor reordering fails to include lower-ranked items from certain categories, leading to those categories being underrepresented in the final recommendation list. Instead, our approach separates coverage from scoring via a two-step procedure: (i) a coverage-first step that explicitly secures items from underrepresented categories in the candidate pool, followed by (ii) a category-aware fill that completes the list with adjusted scores conditioned on the running category histogram. This design goes beyond penalty-only reranking and widens the attainable diversity range while preserving accuracy. Algorithm 1 shows the overall process of coverage-prioritized reranking.

**Algorithm 1 Coverage-prioritized reranking.**

**Input:** recommendation score ${\hat{y}}_{u,i}$ of user *u* for each item *i* in the candidate item set ${ℐ}_{u}^{-}$, category *c*(*i*) for each item *i* in the item set ${ℐ}_{u}^{-}$, the number *k* of recommendation, the number $\hat{k}$ of candidate items, and hyper-parameters $\left\{\lambda_{1},\lambda_{2}\right\}$ controlling the degree of reranking

**Output:** top-*k* recommendation list ${ℛ}_{u}^{k}$ for user *u*

1: ${ℛ}_{u}^{k}=\emptyset$

2: ${\hat{R}}_{u}^{\hat{k}}=\{i|i\in {ℐ}_{u}^{-},\text{ and }{\hat{y}}_{u,i}\text{ is among the largest }\hat{k}\text{ values}\}$

3: ${𝒞}_{u}^{\hat{k}}=\{c_{1},c_{2},\ldots ,c_{m}\}$ such that *c*_*j*_ is a unique category in ${\hat{R}}_{u}^{\hat{k}}$ and sorted in ascending order of frequency:

$\left\{i|i\in {\hat{R}}_{u}^{\hat{k}},c(i)=c_{j}\}|\leq |\{i|i\in {\hat{R}}_{u}^{\hat{k}},c(i)=c_{j+1}\}\right|$

// select one item from each of the least popular categories

4: **for** each category $c\in {𝒞}_{u}^{\hat{k}}[:\lambda_{1}\times k]$ **do**

5:   select an item i^*^ such that $i^{*}=\arg\max_{i\in {\hat{R}}_{u}^{\hat{k}}\text{ and }c(i)=c}\;{\hat{y}}_{u,i}$

6:   ${ℛ}_{u}^{k}\leftarrow {ℛ}_{u}^{k}\cup \{i^{*}\}$

7:   ${\hat{R}}_{u}^{\hat{k}}\leftarrow {\hat{R}}_{u}^{\hat{k}}⧵\{i^{*}\}$

8: **end for**

// iteratively fill the recommendation list considering the penalized score of candidate items

9: **while** $|{ℛ}_{u}^{k}|<k$ **do**

10:   for $i\in {\hat{R}}_{u}^{\hat{k}}$ select the item with the highest adjusted score considering category: $i^{*}=\arg\max_{i\in {\hat{R}}_{u}^{\hat{k}}}{\hat{y}}_{u,i}-\lambda_{2}\times |\{i|i$ $\in {ℛ}_{u}^{k},\;c(i)=c\}|$

11:   ${ℛ}_{u}^{k}\leftarrow {ℛ}_{u}^{k}\cup \{i^{*}\}$

12:   ${\hat{R}}_{u}^{\hat{k}}\leftarrow {\hat{R}}_{u}^{\hat{k}}⧵\{i^{*}\}$

13: **end while**

14: ${ℛ}_{u}^{k}\leftarrow$ sort($\left\{i|i\in {ℛ}_{u}^{k}\right\}$ by ${\hat{y}}_{u,i}$ in descending order)

15: **return** the final recommendation list ${ℛ}_{u}^{k}$

First, we select one item from each of the least popular categories to ensure they are represented and to enhance category coverage. This approach is necessary because items from least popular categories are unlikely to be included in the top-*k* recommendations if we consider only preference scores or apply minimal reranking because their preference scores tend to be lower than those of items in popular categories. In line 3, we identify the unique categories within the candidate items ${\hat{R}}_{u}^{\hat{k}}$ and then sort the categories in ascending order of their frequency. From line 4 to 8, we repeatedly select the highest-scoring item from one of the least popular categories until the desired number of items has been selected. Specifically, given a set ${𝒞}_{u}^{\hat{k}}=\{c_{1},c_{2},\ldots ,c_{m}\}$ of categories such that *c*_*j*_ is a unique category in ${\hat{R}}_{u}^{\hat{k}}$ and sorted in ascending order of frequency in ${\hat{R}}_{u}^{\hat{k}}$, an item i^*^ with the highest prediction score from each category *c* is selected:

$$i^{*}={\arg\max}_{i\in {\hat{R}}_{u}^{\hat{k}}\text{ and }c(i)=c}\;{\hat{y}}_{u,i}$$ (9)

The selected item is added to the final recommendation list ${ℛ}_{u}^{k}$. The number of items to be selected in this phase is $\lambda_{1}\;\times \;k$, where $0<\lambda_{1}<1$ is a hyper-parameter to adjust the level of coverage. Higher the $\lambda_{1}$, the more focus on category coverage is given.

Next, from line 9 to 13 we iteratively fill the recommendation list ${ℛ}_{u}^{k}$ with top-ranked items until reaching the desired number *k*. During each iteration, we penalize the scores of candidate items based on the category frequency of the items already present in ${ℛ}_{u}^{k}$. In line 10, we apply a penalty to the scores of the candidate items for reranking based on category frequency of items already added to ${ℛ}_{u}^{k}$:

$$i^{*}=\arg\max_{i\in {\hat{R}}_{u}^{\hat{k}}}{\hat{y}}_{u,i}-\lambda_{2}\times |\{i|i\in {ℛ}_{u}^{k},\;c(i)=c\}|,$$ (10)

where $\lambda_{2}$ is a hyper-parameter that controls the degree of penalty. A high $\lambda_{2}$ assigns stronger penalties to already represented categories, thereby increasing the overall diversity. Both $\lambda_{1}$ and $\lambda_{2}$ are extendable to user-specific forms, allowing personalized control over diversity within the reranking phase. In line 11, we add the highest-scoring item to the recommendation list, and in line 12, we remove it from the candidate set. This process is repeated until the recommendation list is fully populated.

Finally, in line 14, we reorder the items based on their original predicted scores before returning the final recommendation list. While diversity is not dependent on the order of the recommended items, accuracy metrics (e.g., nDCG) are, thus reordering according to the original predicted scores is crucial for maintaining a high level of accuracy.

This reranking operates on a small candidate set (top-$\hat{k}$ items) and scales linearly with $\hat{k}$, adding negligible latency to the overall pipeline. Hence, category-prioritized reranking is easily deployed in large-scale or high-throughput recommendation environments.

## Experiments

In this section, we perform experiments to answer the following questions:

Q1. **Performance.** How well does CatDive perform in terms of accuracy and diversity compared to other models?
Q2. **Generality.** Is CatDive applicable to various recommendation models, and how does it affect their performance?
Q3. **Ablation Study.** How does each component of CatDive affect the performance?
Q4. **Case Study.** How diverse is the recommendation of CatDive compared to that of non-diversified model?

### Experimental setup

We introduce our experimental setup including datasets, evaluation protocol, baseline approaches, and evaluation metrics.

#### Datasets.

We use three real-world rating datasets as summarized in Table 2. All datasets are publicly released by their providers, and we use them under the terms of their respective licenses. Amazon Books and Amazon Kindle are constructed from Amazon, an online store. We use the first subcategory of items as the category. GoodReads Romance is constructed from GoodReads, a book review site. The subcategories of Romance category are used as the category.

**Table 2:** Summary of datasets.

Dataset	Users	Items	Interactions	Categories
Amazon Books^1^	8,685	9,053	1,043,390	26
Amazon Kindle^1^	8,530	10,057	438,541	17
GoodReads Romance^2^	4,864	4,964	608,816	44

^1^ https://nijianmo.github.io/amazon/index.html

^2^ https://mengtingwan.github.io/data/goodreads.html

#### Baselines.

We compare the performance of CatDive with both diversified and non-diversified methods:

- **Non-diversified Methods:**
  – **GRU4Rec [6].** An RNN-based traditional sequential recommendation model which utilizes a session-parallel mini-batch training process and adopts a pairwise ranking loss function.
  – **SASRec [12].** The state-of-the-art model of traditional sequential recommendation which uses a self-attention mechanism, capturing both short and long-term preferences from users’ past interactions.
- **Diversified Methods:**
  – **MCPRN [20].** A session-based recommendation model that captures multiple purposes within a single session by dynamically routing information through different channels based on the predicted purposes of user interactions. As claimed in the original paper, MCPRN boosts both accuracy and diversity.
  – **MMR [19].** A greedy algorithm to re-rank items provided by a backbone model using the formula (relevance score$\;+\;\lambda_{m}\times \text{diversification score}$); a higher $\lambda_{m}$ leads to higher category diversity and lower accuracy. We use SASRec as the backbone model of MMR for the best performance.
  – **ComiRec [7].** A sequential recommendation framework that consists of two modules: a multi-interest module and an aggregation module. The multi-interest module identifies multiple interests from user behavior sequences, which can be used to retrieve candidate items from a large-scale item pool. The aggregation module reranks the recommendation from the multi-interest module using a controllable factor $\lambda_{c}$ to balance the accuracy and diversity of the recommendations; a higher $\lambda_{c}$ leads to higher category diversity and lower accuracy.

#### Backbone model.

We leverage the state-of-the-art model of sequential recommendation, SASRec, as the backbone model of CatDive.

#### Evaluation metrics.

We employ *leave-one-out* protocol [21,22] where one of each user’s interactions is removed for test. For each listed dataset, we select the last instance from each user’s list of instances, which is ordered by timestamp, to use as the test set. We evaluate the performance of methods in two ways: accuracy and category diversity. Accuracy is used to compare the predicted rank of recommended items to their true rank, and category diversity is used to evaluate how diverse the categories of recommended items are. We set the number *k* of recommendation to 10 and 20 for all datasets. We evaluate the performance with the following four metrics:

- **HitRate@*k* (HR@*k*)**: The proportion of ground-truth items found in the recommendation when *k* items are recommended to each user. HR@*k* considers all items ranked within the first *k* to be equally important. It ranges from 0 to 1 and the accuracy of recommendation is the highest when it is 1.
- **nDCG@*k*:** The cumulative gain of a set of results by summing up the total relevance of each item in the recommendation list when *k* items are recommended to each user. nDCG@*k* considers higher ranks more importantly by monotonically increasing the discount factor. It ranges from 0 to 1 and the accuracy of recommendation is the highest when it is 1.
- **ILD@*k*:** The average inter-list distance of recommendation when *k* items are recommended to each user, which is defined in Eq (1). It ranges from 0 to 1 and the diversity of recommendation is the highest when it is 1.
- **cov@*k*:** The average number of categories recommended to each user relative to the total number of categories when *k* items are recommended to each user as defined in Eq (2). It ranges from 0 to 1 and the diversity of recommendation is the highest when it is 1.

### Performance

**Fig 3:**
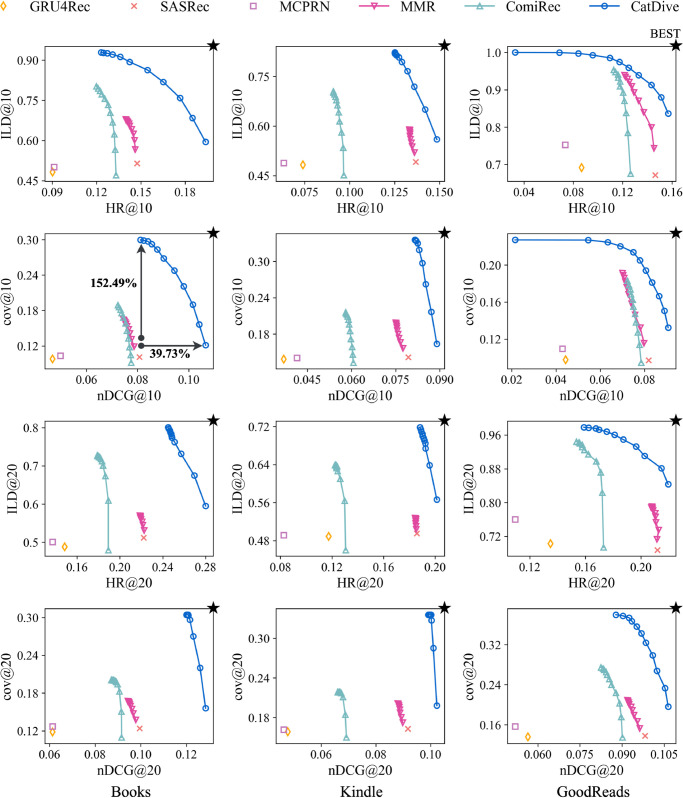
Performance of CatDive and competitors. CatDive is the closest to the best point with the highest category diversity and accuracy among all datasets.

We compare the performance of CatDive and competitors on three real-world datasets in Fig 3. For the diversified methods with reranking (MMR, ComiRec, and the proposed CatDive), we adjust two hyper-parameters $\{\lambda_{1}$, $\lambda_{2}\}$ for coverage-prioritized reranking in increments of 0.1, ranging from 0 to 1, and mark all points. When $\{\lambda_{1}$, $\lambda_{2}\}$ equal to 0, reranking is not applied and diversity is at the lowest point. The category weight *α* is set to 1.0, 0.5, and 0.9 for the best performance, while the confidence weight *β* is set to 0.6, 0.4, and 0.8. The best point with the highest accuracy and the highest diversity in each plot is marked as a black star. Note that CatDive achieves the highest category diversity with least accuracy drop compared to others baselines considering both ILD and coverage. CatDive shows up to 152.49% higher category diversity in the similar level of accuracy and up to 39.73% higher accuracy in the similar level of category diversity.

### Generality

**Fig 4:**
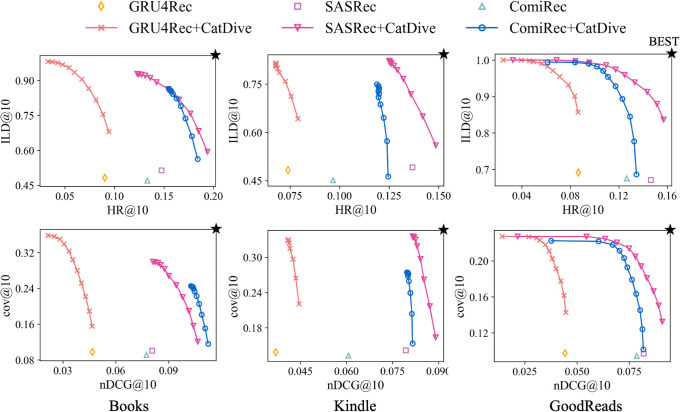
Performance of CatDive applied to different backbone models. CatDive improves both category diversity and accuracy when applied to all models.

To demonstrate the general applicability of CatDive, we apply it to various backbone models and present the performance comparison in Fig 4. We use GRU4Rec, SASRec, and the multi-interest module of ComiRec as the backbone models. GRU4Rec and SASRec are single-interest sequential recommendation models, whereas ComiRec operates as a multi-interest model. For GRU4Rec, *α* is set to 1, while *β* is adjusted to 0.1, 0.4, and 0.3 for Books, Kindle, and GoodReads datasets, respectively. For ComiRec, {α,β} are set to {0.2,0.1}, {0.3,0.01}, and {0.2,0.01} for the same datasets. Note that CatDive consistently enhances both category diversity and accuracy across all backbone models.

### Ablation study

We analyze the effect of multi-embedding and preference-confidence based negative sampling in the training phase. We have three observations from the result in Table 3. First, multi-embedding significantly improves the accuracy of recommendation showing up to 31.35% improvement. Second, preference-confidence based negative sampling dramatically increases category diversity without sacrifice of accuracy, showing up to 47.89% increase with up to 7.06% increase in accuracy. Third, applying both multi-embedding and preference-confidence based negative sampling successfully balances category diversity and accuracy showing up to 36.49% increase in category diversity with up to 32.09% improvement in accuracy.

**Table 3:** Ablation study of multi-embedding (ME) and preference-confidence based negative sampling (NS). ‘Improv.’ next to each component indicates the improvement relative to the original backbone model. The best is marked bold and the second best is underlined.

Dataset	Metric	Original (SASRec)	+ME	Improv.	+NS	Improv.	CatDive (ME&NS)	Improv.
Books	HR@10	0.1475	0.1933	+31.05%	0.1553	+5.29%	**0.1938**	+31.39%
	nDCG@10	0.0807	0.1060	+31.35%	0.0851	+5.45%	**0.1066**	+32.09%
	ILD@10	0.5146	0.4726	–8.16%	**0.6010**	+16.79%	0.5947	+15.57%
	cov@10	0.1010	0.0995	–1.49%	**0.1273**	+26.04%	0.1213	+20.10%
Kindle	HR@10	0.1367	0.1467	+7.32%	0.1434	+4.90%	**0.1485**	+8.63%
	nDCG@10	0.0793	0.0841	+6.05%	0.0849	+7.06%	**0.0890**	+12.23%
	ILD@10	0.4917	0.4742	–3.56%	**0.5772**	+17.39%	0.5597	+13.83%
	cov@10	0.1417	0.1373	–3.11%	**0.1714**	+20.96%	0.1638	+15.60%
GoodReads	HR@10	0.1464	**0.1688**	+15.30%	0.1408	–3.83%	0.1569	+7.17%
	nDCG@10	0.0819	**0.0956**	+16.73%	0.0817	–0.24%	0.0907	+10.74%
	ILD@10	0.6717	0.6780	+0.94%	**0.8532**	+27.02%	0.8368	+24.58%
	cov@10	0.0970	0.0965	+0.52%	**0.1386**	+42.89%	0.1324	+36.49%

### Case study

To verify the category diversity of CatDive, we compare the recommendation results created by CatDive and other model in real-world datasets. Table 4 shows the result from Amazon Books dataset by CatDive and vanilla SASRec. We have two observations. First, given the same user’s history, CatDive recommends books of diverse categories while vanilla SASRec recommends all books from a single category that appears frequently in the user’s history. For instance, vanilla SASRec recommends books only from *Romance* for user 1 while CatDive recommends books from *Science Fiction & Fantasy*, *Literature & Fiction*, *Humor*, and *Romance*. Second, CatDive finds the ground-truth items that are not in the dominant category for each user. For example, the ground-truth item for user 1 is ‘Blood Fury’ in *Science Fiction & Fantasy* category which is not the dominant category of the user 1. This shows that considering category diversity by CatDive helps correctly selecting the target item, preventing the popularity bias of the existing model.

**Table 4:** Recommendation by CatDive and SASrec in Amazon books. CatDive recommends books of diverse categories while SASRec recommends only a single category. *SF & Fan* and *Lit & Fic* stand for *Science Fiction & Fantasy* and *Literature & Fiction*, respectively. The ground-truth items are marked in bold.

user	CatDive		SASRec	
	Title	Category	Title	Category
1	**Blood Fury**	**SF & Fan**	Rigid	Romance
	The Billionaire Takes All	Lit & Fic	Lovestruck	Romance
	With This Man	Humor	69 Million Things I Hate About You	Romance
	Razing Grace	Lit & Fic	Pretty Broken Dolls	Romance
	Just an Illusion	Romance	For the Heart of an Outlaw	Romance
2	Savor You	Lit & Fic	Sweet Nothings	Lit & Fic
	**Blood Red Road**	**SF & Fan**	Break Me	Lit & Fic
	Heart of a Thief	Romance	Rocky Mountain Heat	Lit & Fic
	Cocky Biker	Lit & Fic	The Queen and the Cure	Lit & Fic
	The Queen and the Cure	Lit & Fi	Dear Bridget, I Want You	Lit & Fic
3	**Confessed**	**Lit & Fic**	Something So Right	Romance
	Immortal Op	Romance	FILTHY	Romance
	Burning Bond	Romance	Jarek	Romance
	Perfume Therapy	Lit & Fic	Immortal Ops	Romance
	Take Me With You	Mystery	Burning Bond	Romance

## Conclusion

We propose CatDive, a simple yet effective approach for category diversified sequential recommendation. During training, CatDive integrates category information into the recommendation model by exploiting a multi-embedding approach, which combines item and category embeddings. Additionally, CatDive enhances category diversity by giving more weights to users’ preferred categories when selecting negative samples, while still prioritizing high-confidence negatives based on item popularity to maintain accuracy. Finally, we boost and regulate category diversity by prioritizing underrepresented categories in the reranking phase, using a tunable hyper-parameter. As a result, CatDive demonstrates the state-of-the-art performance on real-world datasets, offering up to 152.49% greater category diversity at similar levels of accuracy and up to 39.73% higher accuracy at comparable levels of category diversity compared to the leading competitor.
